# The B-cell-autoantibody axis in lung cancer immunity

**DOI:** 10.7150/thno.131046

**Published:** 2026-03-03

**Authors:** Yan Huang, Haoyue Hu, Ruoyu Sun, Yi Wang

**Affiliations:** 1 College of Physical Education and Health Science, Yibin University, 8 Jiusheng Road, Cuiping District, Yibin, Sichuan 644000, China.; 2 Department of Oncology, The Third People's Hospital of Yibin, Yibin, Sichuan, China.; 3 Sun Yat-sen University Cancer Center, State Key Laboratory of Oncology in South China, Sun Yat-sen University, Guangzhou, Guangdong 510060, China.; 4 School of Mechanical Engineering, Sichuan University of Science & Engineering, Yibin, Sichuan, China.; 5 Department of Radiation Oncology, Sichuan Clinical Research Center for Cancer, Sichuan Cancer Hospital and Institute, Sichuan Cancer Center, Affiliated Cancer Hospital of University of Electronic Science and Technology of China, Chengdu, China.

**Keywords:** non-small cell lung cancer, tumor-infiltrating B lymphocytes, autoantibodies, tumor microenvironment, tertiary lymphoid structures, immunotherapy

## Abstract

Non-small cell lung cancer (NSCLC) remains a leading cause of cancer-related mortality, and while immune checkpoint inhibitor (ICI) has transformed treatment, resistance remains a critical challenge. Beyond the T-cell-centric view, tumor-infiltrating B lymphocytes (TIL-Bs) and tertiary lymphoid structures (TLSs) have emerged as pivotal prognostic determinants; however, the mechanistic interplay within the B-cell-autoantibody axis remains underexplored. Unlike previous reviews that primarily catalogue B-cell abundance, this synthesis integrates emerging evidence from single-cell RNA sequencing (scRNA-seq) and spatial transcriptomics to dissect the spatiotemporal dynamics of B-cell subsets. We elucidate how the maturation status of TLSs dictates the functional plasticity of TIL-Bs, switching between anti-tumor effector phenotypes (e.g., antibody-secreting plasma cells) and pro-tumor regulatory roles (e.g., IL-10^+^ regulatory B cells). Furthermore, we systematically examine the dualistic role of autoantibodies—not merely as serological biomarkers but as active regulators of the tumor immune microenvironment (TIME) through complement activation and antibody-dependent cell-mediated cytotoxicity (ADCC). Finally, we highlight the clinical and translational implications of targeting this axis, proposing precision strategies such as B-cell-based vaccines and the modulation of TLS neogenesis to overcome ICIs resistance. This review provides a comprehensive roadmap for integrating B-cell biology into next-generation personalized immunotherapy for NSCLC.

## Introduction

Non-small cell lung cancer (NSCLC) is one of the most prevalent malignancies worldwide and continues to present a significant public health challenge due to its high incidence and mortality rates 1. In recent years, immune checkpoint inhibitor (ICI)-based therapies have transformed modern cancer treatment, with particularly notable advances achieved in NSCLC 2, 3. However, the prognosis for the majority of patients remains poor, highlighting an urgent need for novel therapeutic strategies 4, 5. In this context, a comprehensive understanding of the tumor immune microenvironment (TIME) in NSCLC—particularly the roles of tumor-infiltrating B lymphocytes (TIL-Bs) and the autoantibodies they produce—has emerged as an area of intense research interest. The immunoregulatory functions of TIL-Bs and their associated autoantibodies in NSCLC have garnered widespread attention 6, 7. These autoantibodies not only directly target tumor cells but also contribute to anti-tumor immunity by modulating other components of the immune microenvironment, such as T cells and macrophages 8, 9. While recent seminal reviews have significantly advanced our understanding of B-cell biology in cancer, key gaps remain. For instance, Fridman et al elegantly described the "intratumoral immunity cycle" driven by B cells within tertiary lymphoid structures (TLSs) 10, and Yang et al comprehensively summarized the dualistic pro- and anti-tumor functions of B cells 11. However, a unified synthesis that specifically integrates the local spatial dynamics of TIL-Bs with the systemic implications of the autoantibody response in NSCLC is lacking. Most existing literature treats TIL-Bs and autoantibodies as separate entities, overlooking their mechanistic connectivity. To address this gap, this review focuses on the "B-cell-autoantibody axis". We leverage emerging evidence from single-cell and spatial transcriptomics to dissect how the maturation status of TLSs dictates B-cell trajectories. Distinct from broad pan-cancer reviews, we specifically synthesize the clinical utility of this axis in NSCLC, linking biological mechanisms to actionable therapeutic strategies, such as CAR-B cells and biomarker-guided immunotherapy.

## 1. Roles of B lymphocytes within the immune microenvironment of NSCLC

The TIME consists of a variety of immune cells (such as T cells, B cells, macrophages, and dendritic cells), non-immune cells (such as tumor-associated fibroblasts and vascular endothelial cells), extracellular matrix components, cytokines, chemokines, and other signaling molecules 12-14. This microenvironment not only supports tumor growth, invasion, and metastasis but also serves as a critical site where immune cells recognize and mount attacks against tumor cells 15. Immune cells engage in complex interactions with tumor cells within this environment, which can either suppress tumor growth and spread or be exploited by the tumor to evade immune surveillance 12. Therefore, an in-depth understanding of the TIME is of great importance for developing novel immunotherapeutic strategies and improving the efficacy of existing treatments 16, 17. In the complex immune microenvironment of NSCLC, B lymphocytes have emerged as prominent regulators, revealing diverse roles in modulating immune responses and mediating anti-tumor activity 7, 18, 19. These cells contribute to a sophisticated immune regulatory network through mechanisms such as secreting tumor antigen-specific antibodies, modulating the activity of T cells and other immune cells, establishing long-term tumor-specific immune memory, and releasing cytokines that shape the immune microenvironment 20, 21. Collectively, these functions not only enhance the immune system's anti-tumor efficacy but also provide a critical biological foundation for the development of novel immunotherapeutic strategies targeting NSCLC. As our understanding of the roles of B lymphocytes in tumor immunity deepens, they are increasingly recognized as promising therapeutic targets for improving the prognosis of NSCLC patients and advancing the field of immunotherapy.

### 1.1 Basic functions and immunoregulatory roles of TIL-Bs

B lymphocytes play a complex and critical role in the immune microenvironment of NSCLC, significantly influencing tumor immune surveillance and therapeutic responses through their multidimensional functions and mechanisms of action 7, 22. One of their basic functions is to differentiate into plasma cells upon recognizing specific antigens and produce antibodies that specifically bind tumor antigens 23, 24. These antibodies not only directly neutralize pathogens but also indirectly attack tumor cells by promoting antigen presentation, activating the complement system, and enhancing antibody-dependent cell-mediated cytotoxicity (ADCC) 25. In addition, B cells secrete both immunosuppressive cytokines, such as IL-10 and IL-35, and immunostimulatory cytokines, such as IL-6, TNF-α, and IL-12, forming a bidirectional immune regulatory network 26-29. These cytokines act in concert to finely regulate T cell responses: stimulatory factors (e.g., IL-6/IL-12) promote Th1 cell differentiation and CTL activation, thereby enhancing anti-tumor immune responses; inhibitory factors (e.g., IL-10 and IL-35) function by downregulating costimulatory molecules on antigen-presenting cells and directly inhibiting the proliferation of effector T cells. Specifically, IL-10 plays a pivotal role in maintaining immune tolerance by promoting the differentiation of FoxP3+ regulatory T cells (Tregs) and suppressing pro-inflammatory Th1/Th17 responses, thereby preventing immunopathological damage 30, 31. This dynamic balance not only maintains anti-tumor immune activity but also prevents excessive inflammatory responses, providing dual protection in shaping an immune microenvironment that suppresses tumor growth 32. Within the TIME, interactions between TIL-Bs and TLSs establish a complex regulatory network that profoundly influences tumor immune surveillance and the orchestration of anti-tumor immune responses 22. TLSs serve as highly organized centers of immune activation, mimicking the structural architecture and functional compartmentalization of secondary lymphoid organs 33, 34. They are composed of aggregated B cell follicles (often containing germinal centers), T cell zones rich in T cells and dendritic cells (DCs), a supportive stromal network including fibroblastic reticular cells (FRCs) and follicular dendritic cells (FDCs), and characteristic high endothelial venules (HEVs) 35.

**Figure 1:**
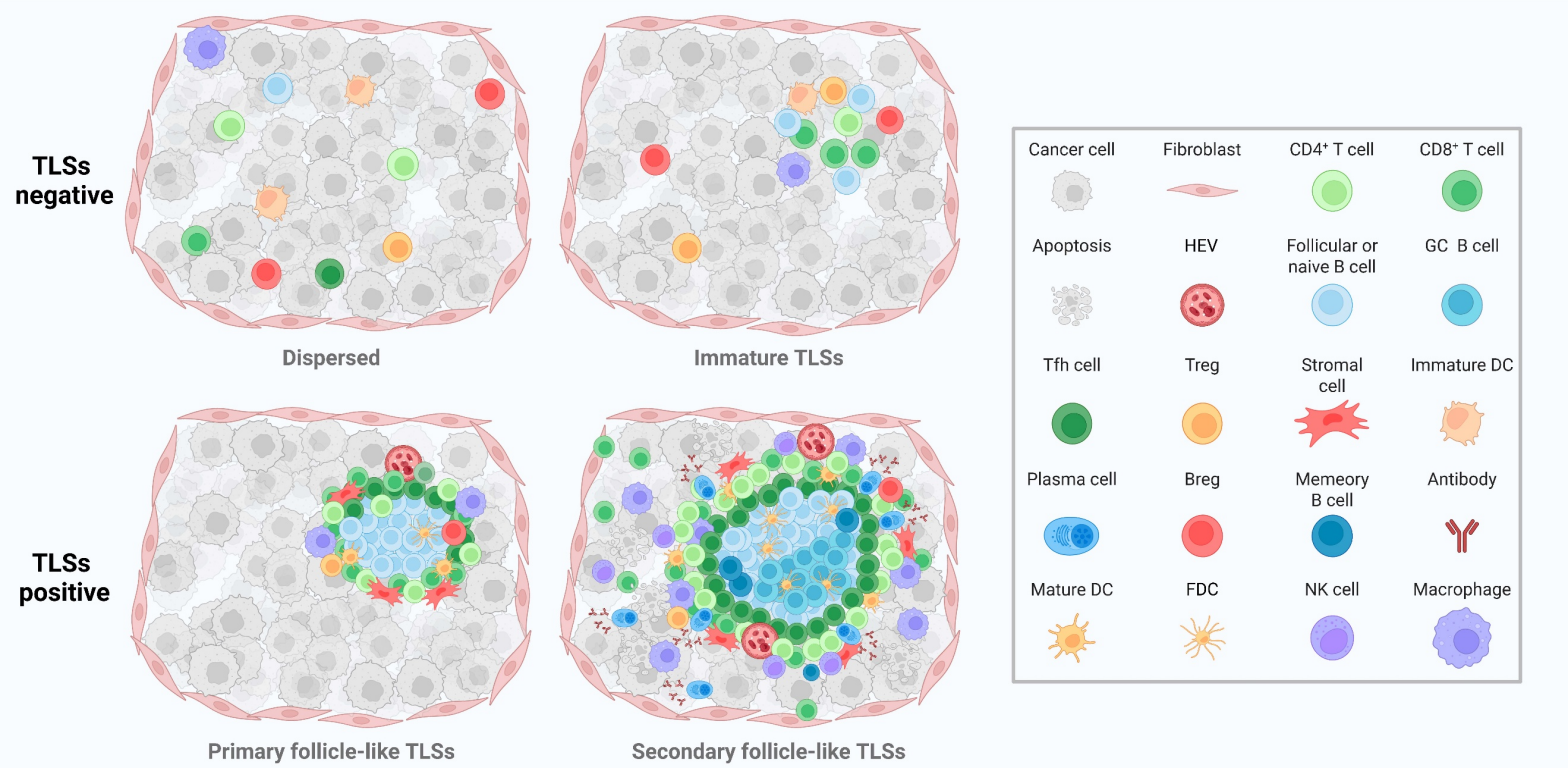
** Structural progression and immune landscape of tumor-associated TLSs.** Dispersed structures are TLSs-negative regions where immune cells such as CD4⁺ T cells, CD8⁺ T cells, macrophages, and Tregs infiltrate loosely without spatial organization. Immature TLSs show early clustering of follicular B cells, DCs, and T cells, but lack GC and stromal scaffolds. Primary follicle-like TLSs are partially organized, featuring follicular B cells, CD4⁺ T cells, Tregs, stromal cells, and HEVs. Secondary follicle-like TLSs are fully mature, characterized by GC B cells, FDCs, Tfh cells, PCs, Abs, and memory B cells, indicating active humoral responses and enhanced local immunity. TLSs: tertiary lymphoid structures; DCs: dendritic cells; GC: Germinal center; Tregs: regulatory T cells; HEVs: high endothelial venules; FDCs: follicular dendritic cells; Tfh cells: follicular helper T cells; PCs: plasma cells; Abs: antibodies; Bregs: regulatory B cells; NK cells: Natural killer cells.

These components, through precise spatial organization and coordinated chemokine/cytokine signaling, collectively establish a localized immune microenvironment within the tumor that facilitates lymphocyte recruitment, tumor antigen presentation, activation of naïve T and B cells, T/B cell crosstalk, affinity maturation, and antibody class-switch recombination 36, 37. This ultimately leads to the generation of effector T cells and high-affinity antibodies. Mature, structurally well-formed TLSs—particularly those containing germinal centers—are generally associated with more robust anti-tumor immune responses and improved clinical outcomes 38, 39. The maturation of TLSs reflects distinct immunological landscapes and correlates with anti-tumor immune activity in NSCLC (Figure 1). In addition, TLSs, as spontaneously formed lymphoid-like structures within the TIME, recapitulate the immunological functions of secondary lymphoid organs and serve as platforms that enable immune cells to effectively recognize and eliminate tumor cells 40. Within this environment, B cells not only contribute to anti-tumor immunity through classical antibody-mediated responses but also play an essential role in the formation, maturation, and maintenance of TLSs 10. B cell functions within TLSs encompass multiple dimensions, including antigen recognition, antibody production, immune regulation, and support of TLS structural maturation 41. Through antigen-specific recognition via the B cell receptors (BCRs) and subsequent antigen presentation, B cells can activate antigen-specific T cells, thereby enhancing cell-mediated immune responses 42, 43. Furthermore, the differentiation of B cells into antibody-secreting plasma cells is a critical step in the production of high-affinity antibodies targeting tumor antigens. These antibodies can directly neutralize tumor cells or indirectly induce tumor cell lysis by promoting ADCC and complement-dependent cytotoxicity (CDC) 44, 45. In the context of immune regulation, B cells precisely regulate the immune microenvironment by secreting a range of cytokines, including interleukins (ILs) and tumor necrosis factor (TNF). This cytokine-mediated modulation not only influences the recruitment and activation of other immune cells but also contributes to the maturation of TLSs 46. Within TLSs, B cell interactions and cytokine production facilitate the formation and maintenance of their internal architecture, which is critical for the local recruitment, activation, and memory generation of immune cells 47. Within TLSs, B cells undergo processes such as antigen selection, somatic hypermutation (SHM), and class switch recombination (CSR), which collectively enhance their capacity to recognize and respond to tumor antigens 22, 48, 49. SHM and CSR are critical mechanisms in the adaptive immune response of B cells 50-52. By increasing antibody diversity and modulating antibody isotypes, these processes enable B cells to generate highly specific and functionally active antibodies, thereby enhancing their effectiveness in anti-tumor immunity 53.

### 1.2 Characteristics, subpopulations, and functional mechanisms of TIL-Bs

**Figure 2:**
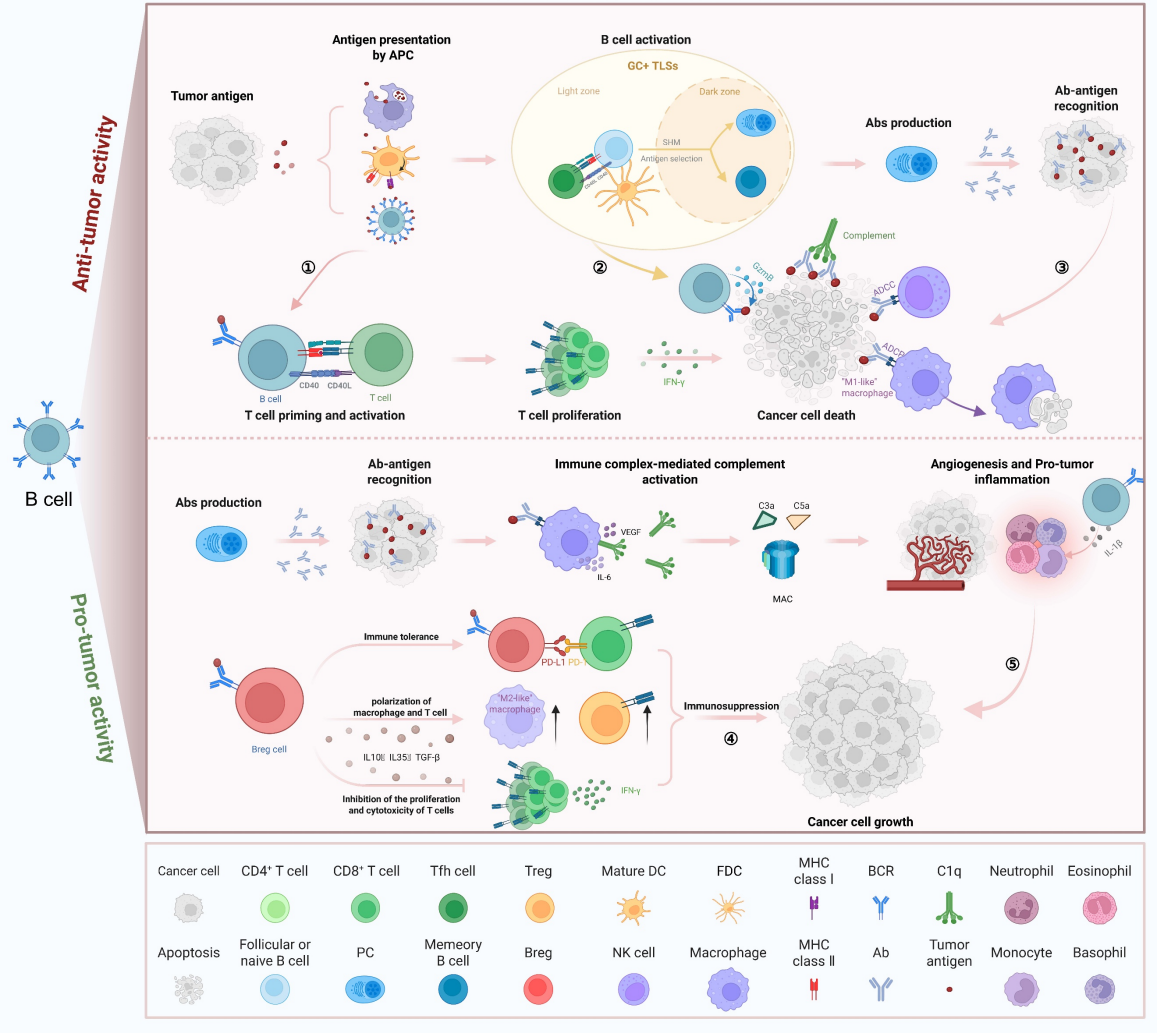
** Dual roles of B cells in tumor immunity.** B cells exert both anti-tumor and pro-tumor effects depending on their localization, activation status, and functional subtype. On the top, activated B cells in GC+ TLSs undergo SHM and CSR, generating high-affinity Abs that promote tumor cell death via ADCC, ADCP, and complement activation. B cells also present antigens to CD4⁺ and CD8⁺ T cells, enhancing cytotoxic T cell responses. On the bottom, Bregs secrete IL-10, IL-35, and TGF-β to inhibit effector T cell proliferation and promote immunosuppressive macrophage polarization. Immune complex formation and cytokine release (e.g., IL-6, VEGF) further drive tumor inflammation, angiogenesis, and immune evasion. GC+ TLSs: germinal centers within tertiary lymphoid structures; CD40: cluster of differentiation 40; CD40L: CD40 ligand; SHM: somatic hypermutation; CSR: class-switch recombination; ADCC: antibody-dependent cell-mediated cytotoxicity; ADCP: antibody-dependent cellular phagocytosis; IL-10: interleukin-10; IL-35: interleukin-35; IL-6: interleukin-6; TGF-β: transforming growth factor-β; VEGF: vascular endothelial growth factor; IFN-γ: interferon-γ; DC: dendritic cell; FDC: follicular dendritic cell; GC: Germinal center; Treg: regulatory T cell; Breg: regulatory B cell; Tfh cell: follicular helper T cell; PC: plasma cell; NK cell: Natural killer cell; Ab: antibody; APC: antigen-presenting cell; GzmB: granzyme B; MAC: membrane attack complex; PD-1: programmed cell death protein 1; PD-L1: programmed death ligand 1; MHC: major histocompatibility complex; BCR: B cell receptor.

B cells are key cellular components of the TIME and are predominantly localized within TLSs. Within the germinal centers of mature TLSs, B cell clones undergo selective activation and clonal expansion, accompanied by CSR and SHM 41. Following this, these B cell clones differentiate into plasma cells capable of producing IgG or IgA antibodies specific to tumor-associated antigens 54-57. In tumors lacking mature TLSs, B cells are either present at low frequencies or differentiate into regulatory phenotypes that secrete immunosuppressive cytokines. Indeed, the abundance of TLSs and B cells varies widely across different tumor types 10. Notably, tumors characterized by mature TLSs, high densities of B cells and plasma cells, and the presence of antibodies targeting tumor-associated antigens are generally associated with more favorable clinical outcomes and improved responses to immunotherapy, compared to tumors lacking these features 58-60. However, B cells exhibit dual roles in the tumor context. In certain cases, their activation may promote the formation of immune complexes, which in turn can induce macrophages and neutrophils to produce pro-inflammatory cytokines 61. Furthermore, in tumors rich in complement components, IgG antibodies can trigger the complement cascade. This activation generates anaphylatoxins (such as C5a) that recruit myeloid suppressor cells, thereby sustaining tumor-promoting inflammation and angiogenesis 62, 63. These findings suggest that B cells can mediate anti-tumor activity through antibody production and antigen presentation, yet may also contribute to inflammation and immune evasion under certain microenvironmental conditions. B cells engage in both pro-tumor and anti-tumor activities via cytokine secretion, antigen presentation, and effector cell modulation (Figure 2). The processes of antigen selection, SHM, and CSR together govern the antigen recognition capacity and antibody diversity of B cells, forming the foundation for their roles in immune responses and memory formation. Through the coordinated action of these mechanisms, B cells are able to generate highly specific and functionally diverse antibodies, thereby defending against pathogen invasion and maintaining immune homeostasis 64. Antigen selection represents an early phase of B cell maturation that takes place in primary lymphoid organs such as the bone marrow. In this stage, B cells engage antigens via their surface BCRs to recognize specific antigenic targets 65. B cells can undergo activation, proliferation, and subsequent differentiation only when their BCRs bind to an antigen and receive the necessary co-stimulatory signals. This selective mechanism ensures that B cells specific to foreign antigens are positively selected and expanded, while self-reactive B cells are eliminated, thereby contributing to the prevention of autoimmune diseases 66. SHM introduces point mutations into the variable regions of the immunoglobulin heavy and light chain genes in B cells. These mutations substantially enhance the structural diversity of antibody binding sites, thereby increasing the affinity of antibodies for their corresponding antigens 67. SHM provides B cells with the opportunity to progressively enhance antibody affinity during the germinal center response, representing a critical step in the production of high-affinity antibodies 68.

**Figure 3:**
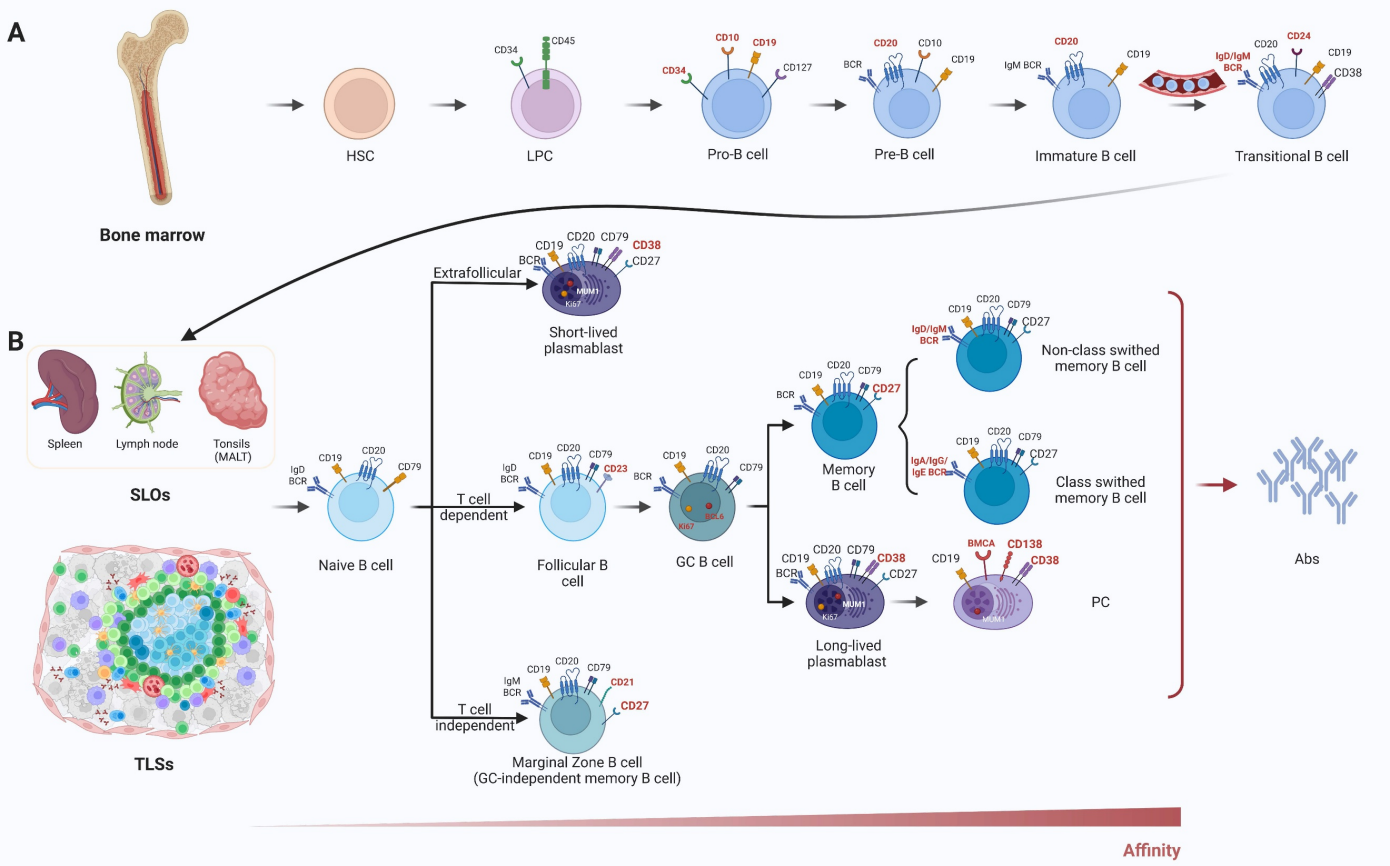
** Development and functional differentiation of B cells.** (A) B cell lineage originates in the bone marrow, progressing through Pro-B, Pre-B, and immature B cell stages. These stages are defined by sequential acquisition of markers such as CD10, CD19, CD20, and surface IgM/IgD. (B) After migrating into SLOs or TLSs, B cells differentiate into various subsets including naïve B cells, memory B cells, plasmablasts, and PCs through T cell-dependent or -independent pathways. Surface markers (e.g., CD27, CD38, MUM1, Ki67) distinguish GC B cells, memory B cells, and Ab-secreting cells. SLOs: secondary lymphoid organs; TLSs: tertiary lymphoid structures; CD: cluster of differentiation; MUM1: multiple myeloma oncogene 1; Abs: antibodies; IgD/IgM/IgA/IgG/IgE: immunoglobulin D/M/A/G/E; BCR: B cell receptor; HSC: hematopoietic stem cell; LPC: lymphoid progenitor cell; Pro-B: progenitor B cell; Pre-B: precursor B cell; GC: germinal center; PC: plasma cell; BMCA: B cell maturation antigen; MALT: mucosa-associated lymphoid tissue.

CSR is a recombination process initiated in activated B cells upon antigen stimulation, enabling the switch of antibody isotypes—for example, from IgM to IgG, IgA, or IgE 69-72. This process modifies the constant region of the immunoglobulin gene without altering the variable region—the portion responsible for antigen specificity—thus preserving specific recognition of the target antigen while altering the antibody's effector function and tissue distribution 69, 73. CSR is regulated by specific cytokine milieus and transcriptional regulatory factors, playing a critical role in enabling B cells to mount appropriate responses to diverse pathogens and modulate immune responses 74. TIL-Bs display distinct phenotypic features, functional classifications, and mechanisms of action within the immune microenvironment of NSCLC, constituting a complex network of interactions between immune surveillance and tumor resistance mechanisms. These cells infiltrate directly into the tumor tissue and establish close associations with tumor cells and other immune components such as T cells and macrophages, forming a multifunctional immune effector front. The phenotypic and functional diversity of B cells in the TIME is determined by their developmental trajectory and activation status (Figure 3).The presence of TIL-Bs not only reflects the host's endogenous immune response to the tumor but also highlights potential targets for immunotherapeutic intervention 75, 76.

## 2. Roles of autoantibodies within the immune microenvironment of NSCLC

Within the complex immune microenvironment of NSCLC, autoantibodies play distinct and functionally significant roles. These antibodies, endogenously produced by the host immune system, can recognize and target normal or altered self-components, including tumor-associated antigens expressed on cancer cells 77-79. Although autoantibodies are typically associated with pathological processes in autoimmune diseases, their presence in malignancies such as NSCLC may indicate an endogenous immune attempt to counteract tumor development 79. These antibodies can recognize and bind to specific antigens expressed on the surface of tumor cells, such as tumor-specific neoantigens or overexpressed self-proteins, and mediate tumor cell lysis and apoptosis through mechanisms including ADCC and complement system activation 80, 81. Another critical role of autoantibodies in NSCLC lies in their potential as biomarkers for early diagnosis, monitoring of disease progression, and assessment of therapeutic efficacy 82, 83. Because certain autoantibodies may be detectable during the early stages of tumorigenesis, they can serve to identify patients with tumors who remain asymptomatic. Moreover, dynamic changes in the autoantibody profile may reflect the biological behavior of the tumor—such as its aggressiveness and clinical prognosis—thereby offering valuable insights to inform clinical decision-making 84, 85. However, the functions of autoantibodies are not uniformly beneficial; in some cases, they may facilitate tumor growth and 86-89. Through specific binding to surface antigens on tumor cells, autoantibodies can modulate tumor growth and progression, either by promoting or inhibiting these processes 58. The functional activity of autoantibodies is determined by factors such as their specific antigen targets, concentration, isotype, and interactions with other components of the immune system 90, 91. For instance, IgG1-type autoantibodies are generally regarded as possessing anti-tumor properties 92. They may function by inducing apoptosis in tumor cells or by enhancing immune recognition and clearance of malignant cells. These antibodies can activate immune effector cells, such as natural killer cells (NKs) and macrophages, thereby promoting the targeted elimination of tumor cells 93, 94. In contrast, autoantibodies of the IgA and IgG3 isotypes may contribute to tumor progression 95, 96. These antibodies can activate specific receptors on tumor cells, initiating signaling pathways that promote cellular proliferation and division, or assist tumor cells in evading immune surveillance, thereby facilitating tumor development 97.

### 2.1 Definition and immunological significance of autoantibodies

Autoantibodies are antibodies generated by the host immune system that specifically target self-antigens—components of the body's own tissues, cells, or molecules 8. Under normal physiological conditions, the immune system maintains self-tolerance through established immunological mechanisms, preventing reactivity to self-antigens. However, in certain circumstances, this tolerance may break down, causing the immune system to erroneously recognize self-tissues as foreign or threatening, thereby leading to the production of autoantibodies 98. This phenomenon is frequently observed in a range of autoimmune diseases, such as systemic lupus erythematosus and rheumatoid arthritis, where autoantibody production is closely linked to disease pathogenesis 99.

From an immunological perspective, the presence of autoantibodies highlights the complexity of the immune system's surveillance and response mechanisms 100. Autoantibodies not only signify an immune response directed against self-tissues but also indicate a disruption of immune tolerance 101, 102. Moreover, their production may represent an immune reaction to endogenous alterations, such as changes in tumor cell surface antigens during the course of tumor development. Tumor cells frequently express aberrant proteins or overexpress otherwise normal self-proteins, which can serve as targets for autoantibodies and thereby initiate an anti-tumor immune response 103-106. Therefore, in the field of oncology, the detection and characterization of autoantibodies are of significant importance. They can serve not only as biomarkers for early-stage tumors but also offer valuable insights into tumor immune evasion mechanisms and inform the development of immunotherapeutic strategies 82. In malignancies such as NSCLC, autoantibodies may arise in response to specific tumor-associated antigens. Their presence not only reflects an endogenous immune attempt to counter tumor development, but also highlights the complexity of immune recognition and anti-tumor immune response mechanisms 107.

### 2.2 Types, functions, and biomarker potential of autoantibodies in NSCLC

In the field of NSCLC research, the identification and application of autoantibodies represent a promising avenue of investigation. These autoantibodies are generated by the patient's immune system in response to tumor-associated autoantigens, and their diversity and functional roles reflect the complex interplay between tumor cells and the host immune system. They can target a wide range of tumor-associated antigens, including tumor-specific mutated proteins, overexpressed self-proteins, and distinct surface molecules expressed by tumor cells 108. On one hand, the presence of these autoantibodies facilitates immune recognition and targeting of tumor cells. By binding to specific antigens on the tumor cell surface, autoantibodies can directly contribute to tumor cell elimination or indirectly induce cell death through mechanisms such as ADCC and complement activation 109. In addition, autoantibodies can enhance tumor recognition and elimination by effector immune cells, such as T cells and natural killer (NK) cells, through the opsonization of tumor cells 110. On the other hand, the emergence and dynamic changes of autoantibodies in NSCLC offer novel biomarker candidates for early diagnosis, therapeutic monitoring, and prognostic evaluation. Specific autoantibody profiles may serve as early indicators of tumor presence and progression, and in some cases, can be detected prior to the manifestation of clinical symptoms, thereby enabling the possibility of early diagnosis 82. Changes in autoantibody levels during the course of cancer therapy can serve as indicators of treatment response and predictors of disease recurrence or progression. Moreover, the presence of specific autoantibodies is closely linked to patient prognosis, as their expression levels may reflect tumor aggressiveness and correlate with overall survival outcomes 58. However, the application of autoantibodies as biomarkers in NSCLC faces several challenges, including their high degree of heterogeneity, the need for rigorous assessment of specificity and sensitivity, and substantial inter-individual variability. Consequently, precise identification of autoantibodies associated with specific tumor processes, along with a comprehensive understanding of their induction mechanisms and functional pathways, is critical for the development of effective diagnostic and therapeutic strategies. In conclusion, research into autoantibodies in NSCLC has revealed their multifaceted roles in tumor immunology and underscored their significant potential as biomarkers for cancer diagnosis and treatment. As our understanding deepens and relevant technologies advance, autoantibodies hold promise as valuable tools in the clinical management of NSCLC.

## 3. Functional roles and underlying mechanisms of TIL-Bs and autoantibodies in NSCLC

In recent years, research on NSCLC has made significant strides in elucidating the roles of TIL-Bs and autoantibodies, laying a critical scientific foundation for understanding the complexity of the TIME and for the development of novel therapeutic strategies 111. These findings have not only elucidated the functional mechanisms of TIL-Bs and autoantibodies in tumor initiation and progression, but also identified novel biomarker candidates for early diagnosis, therapeutic response assessment, and prognostic evaluation in NSCLC. As a critical component of the TIME, research on TIL-Bs has primarily focused on elucidating their roles and mechanisms in tumor immunity. Recent studies have increasingly revealed the complex regulatory functions of B cells and autoantibodies in shaping the TIME. B cells are closely associated with TLSs located adjacent to tumor nests, and their differentiation and functional outcomes are influenced by the maturation status of these TLS 58. In immature TLSs, B cells may differentiate into regulatory B cells (Bregs) that secrete immunosuppressive cytokines and contribute to tumor progression. In contrast, within mature TLSs containing germinal centers, B cells undergo selection, clonal expansion, affinity maturation, and CSR, ultimately differentiating into plasma cells capable of producing anti-tumor antibodies 112. In this context, the presence of anti-tumor antibodies and plasma cells is associated with prolonged patient survival and enhanced responses to immunotherapy. Identifying tumor-specific or overexpressed antigens and distinguishing them from autoantigens induced by ICI treatment remains a major challenge in the development of novel antibody-based immunotherapeutic strategies. In their study, Hao et al. reported that B cell infiltration within the TIME of NSCLC patients is positively correlated with favorable prognosis, suggesting that B cells may represent a promising therapeutic target 49. Furthermore, the role of autoantibodies in NSCLC has garnered increasing attention, with studies demonstrating that the presence of specific autoantibodies is closely linked to disease progression and patient prognosis. For instance, Xu et al. reported the identification of autoantibodies targeting specific antigens that may serve as biomarkers for the early diagnosis of NSCLC 113. However, the role of B cells is not uniformly tumor-suppressive, as their functional heterogeneity within the TIME also allows them to contribute to immune evasion. Cerqueria et al. demonstrated that Bregs can facilitate tumor immune escape through the secretion of immunosuppressive cytokines, such as interleukin-10 (IL-10) 114. In addition, autoantibodies may also interfere with anti-tumor immune responses through the formation of immune complexes. In summary, B cells and autoantibodies exhibit complex and multifaceted roles in NSCLC, contributing both to anti-tumor immunity and to the facilitation of immune evasion. Future studies should aim to elucidate the precise mechanisms by which these cells and molecules operate in NSCLC, thereby informing the development of novel immunotherapeutic strategies.

**Figure 4:**
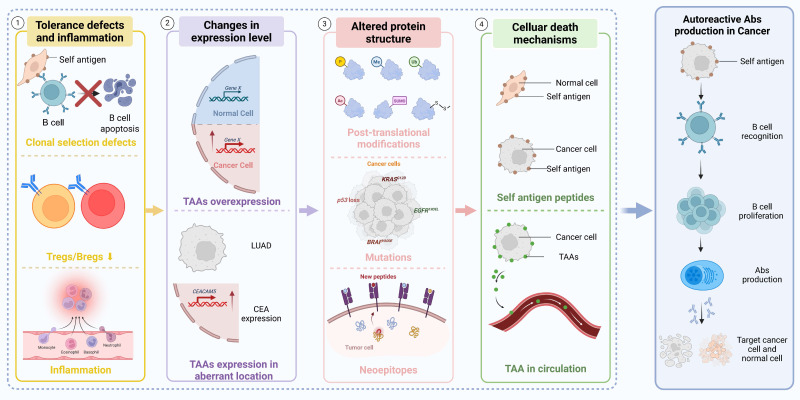
** Mechanisms of autoreactive Abs production in cancer.** Autoreactive Abs production in cancer results from a multistep process involving tolerance defects and inflammation, changes in expression level, altered protein structure, and cellular death mechanisms. Initially, tolerance defects and chronic inflammation lead to the failure of central and peripheral immune checkpoints, allowing the survival of self-reactive B cells. These B cells respond to TAAs that are either overexpressed or ectopically expressed—representing changes in expression level. Additionally, altered protein structure caused by mutations or post-translational modifications exposes neoepitopes that further trigger B cell activation. As cancer cells undergo various forms of cell death, self-antigen peptides are released into circulation, amplifying B cell recognition. These converging mechanisms collectively lead to autoreactive Abs production in cancer, where Abs may bind both tumor and normal antigens. TAAs: tumor-associated antigens; Abs: antibodies; LUAD: lung adenocarcinoma; CEA: carcinoembryonic antigen; P: phosphorylation; Me: methylation; Ub: ubiquitination; Ac: acetylation; SUMO: small ubiquitin-like modifier; -S-S-: disulfide bond.

The immunoregulatory functions of TIL-Bs and autoantibodies in NSCLC are characterized by notable diversity and complexity within the TIME. Research has shown that the number of TIL-Bs is significantly increased in tumor tissues compared to adjacent non-tumor tissues in NSCLC patients. These TIL-Bs are capable of effectively presenting antigens to CD4⁺ tumor-infiltrating lymphocytes (TILs) and can be phenotypically classified into activated and exhausted subsets. Activated TIL-Bs are associated with effector T cell responses, whereas exhausted TIL-Bs are linked to Tregs phenotypes 115, 116. Autoantibodies may affect tumor cell viability through their binding to tumor-associated antigens or modulate immune responses via the formation of immune complexes 8. The generation of autoantibodies is closely linked to the regulatory functions of B cells, particularly the mechanisms of clonal tolerance and clonal redemption. Multiple immunological mechanisms underlie the production of tumor-associated autoantibodies in NSCLC, including defects in tolerance and aberrant antigen expression (Figure 4).

**Figure 5:**
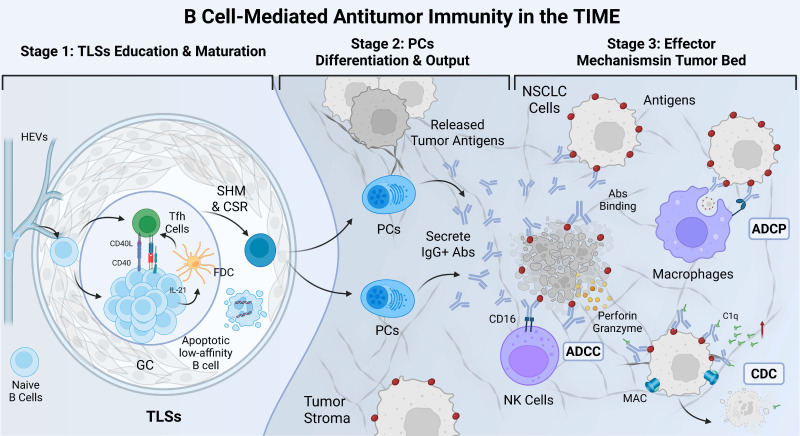
** Mechanistic view of the B-cell-autoantibody-tumor axis mediating antitumor immunity in the NSCLC microenvironment.** This integrative schematic illustrates the sequential progression of the B-cell response along three functional stages. Stage 1 (TLSs Education & Maturation): Within the GC of mature TLSs, naïve B cells interact with Tfh cells and FDCs. Through CD40/CD40L signaling and cytokine support (e.g., IL-21), B cells undergo SHM and CSR to increase antigen affinity and eliminate apoptotic low-affinity clones. Stage 2 (PCs Differentiation & Output): Selected high-affinity B cells differentiate into PCs, which secrete large quantities of tumor-specific IgG+ Abs. Stage 3 (Effector Mechanisms in Tumor Bed): These Abs bind to specific antigens on NSCLC cells, triggering tumor destruction via multiple mechanisms: ADCP by macrophages, ADCC mediated by NK cells (via CD16), and CDC via C1q recruitment and MAC formation. GC: germinal center; Tfh cells: follicular helper T cells; FDCs: follicular dendritic cells; CD40: cluster of differentiation 40; CD40L: CD40 ligand; IL-21: interleukin-21; SHM: somatic hypermutation; CSR: class-switch recombination; PCs: plasma cells; Abs: antibodies; ADCP: antibody-dependent cellular phagocytosis; ADCC: antibody-dependent cell-mediated cytotoxicity; NK cells: Natural killer cells; CDC: complement-dependent cytotoxicity; MAC: membrane attack complex.

These processes maintain a balance between the control of B cell self-reactivity and the initiation of effective immune responses against foreign antigens 117. In addition to antibody-mediated effects, B cells also exert immunoregulatory functions by modulating the activity of T cells and other immune cells, either directly or indirectly, through the secretion of cytokines such as interleukin-10 (IL-10) 118. Bregs play a crucial role in maintaining immune tolerance and preventing autoimmune responses. Their functional roles in NSCLC and other malignancies are increasingly being elucidated through ongoing research 119. In summary, TIL-Bs and autoantibodies exhibit complex and multifaceted roles within the immune microenvironment of NSCLC. They contribute not only to the direct immune recognition and elimination of tumor cells but also modulate immune responses in the TIME, thereby influencing tumor progression and therapeutic outcomes. These insights provide a critical scientific foundation for the development of novel therapeutic strategies. To visually integrate these concepts, we illustrate the complete mechanistic pathway of the B-cell-autoantibody-tumor axis in Figure 5.

A number of clinical studies have evaluated the prognostic value of TIL-Bs in NSCLC, employing diverse detection methods, biomarkers, and patient cohorts. Representative studies have highlighted the relationship between TIL-B infiltration and patient prognosis across different tumor subtypes and clinical stages (Table 1).

Future research should focus on elucidating the precise mechanisms by which TIL-Bs and auto antibodies operate within the NSCLC immune landscape, and on leveraging this knowledge to enhance current treatments or identify new therapeutic targets. To provide clarity, we synthesize the distinct markers and functions of anti-tumor versus pro-tumor B-cell phenotypes in Table 2.

Key challenges include the accurate identification and functional classification of TIL-Bs, the dissection of autoantibody generation mechanisms and their relationship to tumor progression, and the translation of these findings into clinical applications. Moreover, the expression patterns and functional roles of TIL-Bs and autoantibodies may vary widely depending on tumor stage and individual patient characteristics, highlighting the need for increasingly refined and personalized approaches in future investigations.

## 4. Therapeutic applications of TIL-Bs and autoantibodies in NSCLC

**Figure 6:**
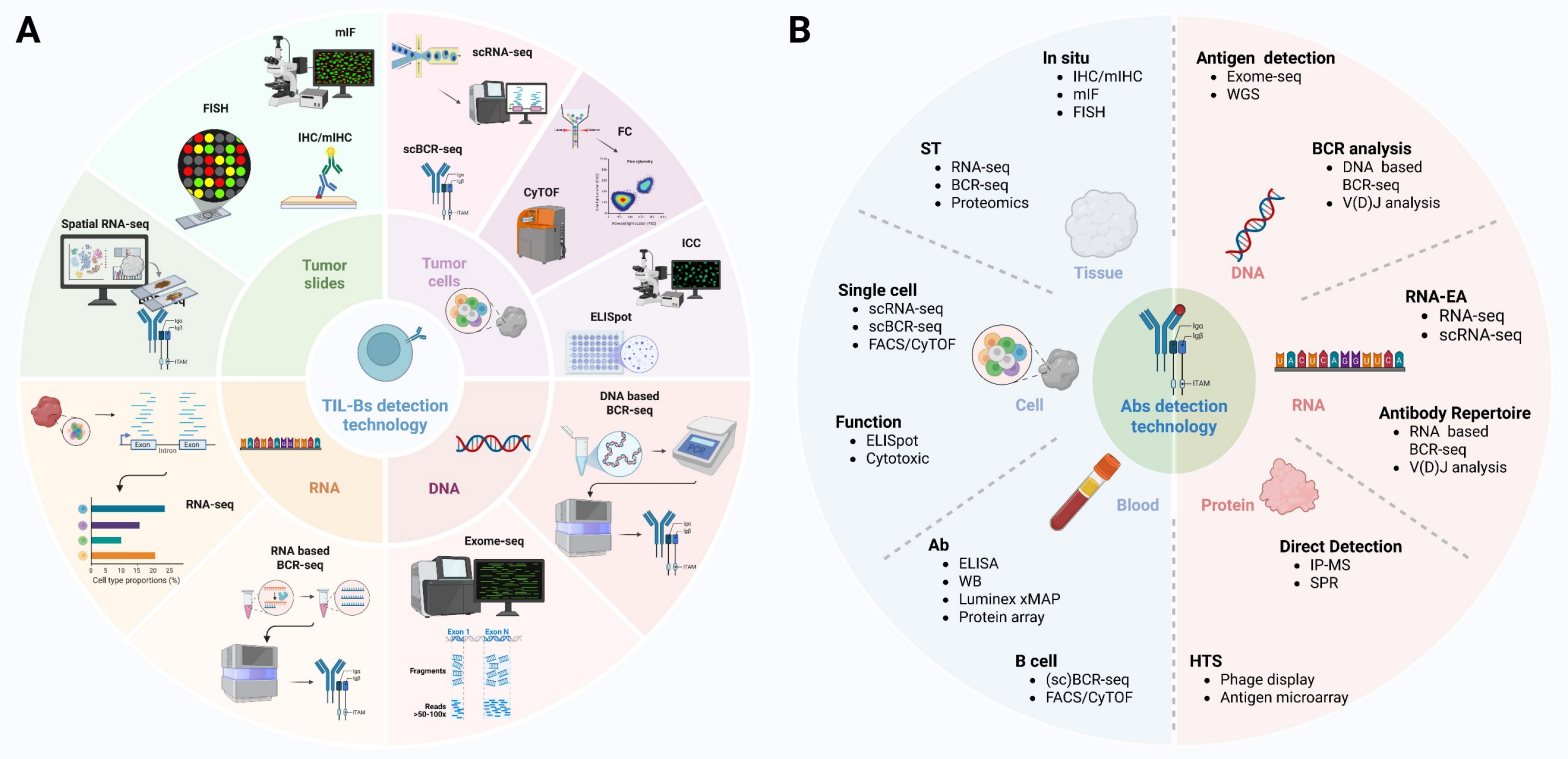
** Comprehensive strategies for TIL-Bs and Abs detection in tumors.** TIL-Bs and Abs detection technology integrate multiple platforms including DNA/RNA-based sequencing, protein-level profiling, and spatial localization approaches. In situ methods such as IHC, mIHC, mIF, and FISH allow visualization of B cell distribution and phenotype within tumor tissues. Bulk and single-cell sequencing approaches—such as RNA-seq, scRNA-seq, BCR-seq, and scBCR-seq—enable transcriptomic and clonal analysis of TIL-Bs, revealing their diversity and antigen specificity. Abs detection technologies including ELISA, protein arrays, IP-MS, and Luminex allow quantification and characterization of secreted Abs. These methodologies, when combined with spatial transcriptomics, CyTOF, and exome-seq, provide a multi-dimensional framework for understanding the functional role of TIL-Bs and tumor-reactive Abs in the tumor microenvironment. TIL-Bs: tumor-infiltrating B lymphocytes; Abs: antibodies; BCR-seq: B cell receptor sequencing; CyTOF: cytometry by time of flight; DNA: deoxyribonucleic acid; ELISA: enzyme-linked immunosorbent assay; ELISpot: enzyme-linked immunospot; Exome-seq: exome sequencing; FACS: fluorescence-activated cell sorting; FC: flow cytometry; FISH: fluorescence in situ hybridization; HTS: high-throughput sequencing; ICC: immunocytochemistry; IHC: immunohistochemistry; IP-MS: immunoprecipitation-mass spectrometry; mIF: multiplex immunofluorescence; mIHC: multiplex immunohistochemistry; RNA-EA: RNA-based expression analysis; RNA-seq: RNA sequencing; scBCR-seq: single-cell B cell receptor sequencing; scRNA-seq: single-cell RNA sequencing; SPR: surface plasmon resonance; ST: spatial transcriptomics; V(D)J analysis: variable (diversity) joining analysis; WB: western blot; WGS: whole genome sequencing; xMAP: multi-analyte profiling.

Therapeutic strategies involving TIL-Bs have primarily focused on their capacity to directly recognize and eliminate tumor cells. Studies have demonstrated that TIL-Bs can facilitate the opsonization and clearance of tumor cells by producing antibodies targeting specific tumor-associated antigens 48, 143. This mechanism plays a particularly critical role in the context of immunotherapy, especially when combined with immune checkpoint inhibitors (ICIs), as it can significantly enhance therapeutic efficacy 144. Augmenting the function of TIL-Bs or increasing their abundance within the TIME can further potentiate tumor-specific immune responses, resulting in enhanced clinical outcomes for patients. Moreover, the antigen-presenting capacity of TIL-Bs offers an additional pathway for activating antigen-specific T cell responses, thereby reinforcing cell-mediated immune clearance mechanisms 145. The application of autoantibodies in the treatment of NSCLC lies primarily in their potential as biomarkers and therapeutic targets. Autoantibody detection can serve not only as a tool for early tumor diagnosis and prognostic assessment, but also as a means to inform the development of individualized therapeutic strategies 79, 146, 147. Moreover, elucidating the mechanisms underlying the interactions between autoantibodies and tumor cells may facilitate the identification of novel therapeutic targets and the development of targeted drugs, thereby offering additional treatment options for patients with NSCLC. Despite the promising potential of TIL-Bs and autoantibodies in NSCLC therapy, several challenges must be overcome to enable their successful clinical translation. Advances in high-dimensional and spatial profiling technologies have facilitated the detection and characterization of TIL-Bs and autoantibodies (Figure 6). These include the precise modulation of TIL-B activity and abundance, the prevention of unintended autoimmune responses, and the assurance of therapeutic safety and efficacy. Furthermore, advancing the use of autoantibodies as therapeutic targets requires addressing key issues of specificity and sensitivity to ensure accurate targeting of tumor cells without affecting normal tissues.

### 4.1 Innovative immunotherapeutic strategies: potential for modulation of TIL-Bs and autoantibodies

**Figure 7:**
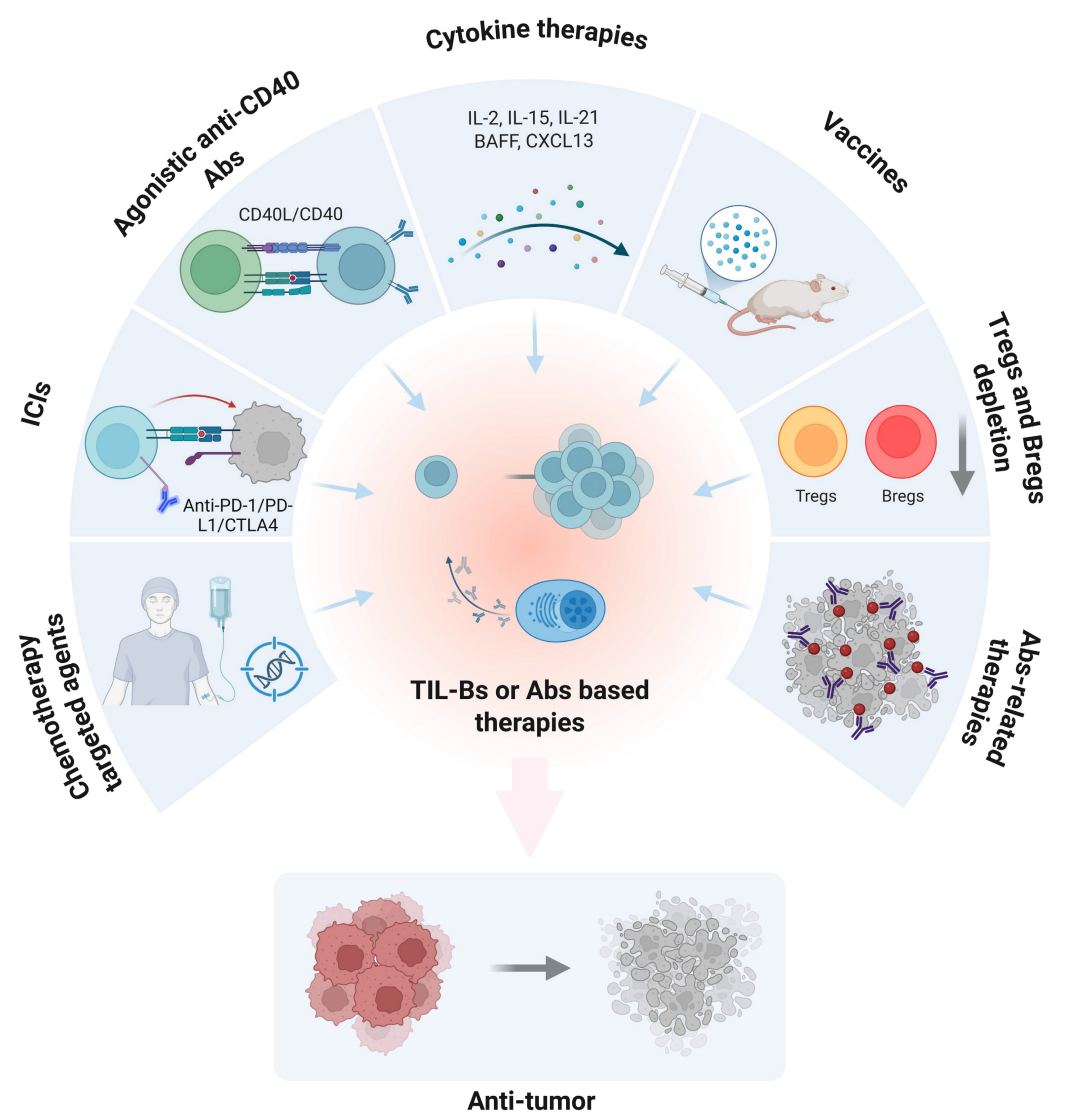
** Therapeutic strategies based on TIL-Bs or Abs responses in cancer.** This schematic illustrates multiple immunotherapeutic approaches that aim to enhance or exploit TIL-Bs or antibody-mediated mechanisms to promote anti-tumor immunity. Clockwise from top left: (1) Agonistic anti-CD40 antibodies activate B cells via CD40/CD40L signaling; (2) Cytokine therapy (e.g., IL-2, IL-15, IL-21, BAFF, CXCL13) supports B-cell proliferation and activation; (3) Cancer vaccines stimulate antigen-specific B cell responses; (4) Depletion of immunosuppressive Tregs and Bregs alleviates immune suppression; (5) Antibody-related therapies directly target tumor cells; (6) Chemotherapy and targeted agents can modulate the tumor microenvironment and affect B-cell responses; (7) Immune checkpoint blockade (e.g., anti-PD-1 Abs) indirectly enhances TIL-B activity. Collectively, these strategies aim to amplify the anti-tumor function of B cells or Abs, leading to tumor regression. TIL-Bs: tumor-infiltrating B lymphocytes; Abs: antibodies; ICIs: immune checkpoint inhibitors; PD-1: programmed cell death protein 1; PD-L1: programmed death ligand 1; CTLA4: cytotoxic t-lymphocyte-associated protein 4; CD40: cluster of differentiation 40; CD40L: CD40 ligand; IL-2: interleukin-2; IL-15: interleukin-15; IL-21: interleukin-21; ; CXCL13: c-x-c motif chemokine ligand 13; BAFF: b-cell activating factor; Tregs: regulatory T cells; Bregs: regulatory B cells.

This can be achieved through multiple strategies, including the use of ICIs to alleviate immunosuppressive constraints on TIL-B function, or the application of vaccine-based approaches to directly stimulate TIL-B responses against specific tumor antigens. Furthermore, the genetic engineering of TIL-Bs to enhance their tumor recognition and cytotoxic potential represents an emerging direction in current research 148, 149. A variety of immunotherapeutic approaches targeting TIL-Bs or harnessing tumor-reactive antibodies are currently under investigation (Figure 7). One approach involves screening for and characterizing autoantibodies that recognize NSCLC-specific antigens, thereby enabling the development of novel passive immunotherapies, such as monoclonal antibody treatments 150. This can be achieved, for example, by using tumor vaccines or immunomodulatory agents to stimulate the generation of specific autoantibodies, thereby potentiating anti-tumor immune responses 58, 151. For instance, detailed profiling of TIL-B phenotypes and autoantibody repertoires within the TIME can enable the customization of immunotherapy regimens tailored to each patient, thereby enhancing therapeutic efficacy while minimizing unnecessary side effects 117, 152.

### 4.2 Challenges and future opportunities in clinical translation

While the translation of research on TIL-Bs and autoantibodies into therapeutic strategies for NSCLC holds significant promise, it also presents substantial challenges. These challenges encompass not only the inherent complexity of the underlying science and technology but also critical issues related to the feasibility and safety of implementing such approaches in clinical practice 58. Nonetheless, these challenges also present valuable opportunities for future advancement. With continued exploration and innovation, it is anticipated that current barriers can be overcome, ultimately enabling the development of more effective therapeutic strategies for patients with NSCLC.

One of the major challenges in this field is to elucidate the precise roles and underlying mechanisms of TIL-Bs and autoantibodies within the immune microenvironment of NSCLC 153. Although accumulating studies have highlighted their critical contributions to shaping anti-tumor immunity, the inherent complexity and heterogeneity of the TIME demand more granular analyses of their context-dependent functions. For instance, the same B-cell subset may exert either anti-tumor or pro-tumor effects depending on the spatial organization of TLSs, the cytokine milieu, and tumor stage 154-157. Furthermore, translating these findings into clinical practice requires the ability to precisely modulate TIL-B and autoantibody activity, while minimizing the risks of autoimmune toxicity and off-target immune activation. Addressing these issues will be pivotal to harnessing TIL-Bs and autoantibodies as reliable therapeutic targets or biomarkers in NSCLC.

A further critical challenge lies in the development of highly sensitive and specific biomarker detection technologies. Although the presence of TIL-Bs and autoantibodies offers promising biomarkers for the diagnosis and therapeutic monitoring of NSCLC, achieving rapid and accurate detection of these markers in clinical settings—particularly at early stages of tumor development—remains a significant technical hurdle 158. Furthermore, ensuring high sensitivity and specificity in biomarker detection is essential for informing the design of personalized treatment strategies. Although significant challenges remain, the potential for future advancement is equally considerable. With the rapid progress in disciplines such as immunology, molecular biology, and bioinformatics, emerging research tools and technologies—including single-cell sequencing, high-throughput antibody screening, and artificial intelligence-driven data analysis—are poised to play a pivotal role in overcoming these obstacles 159, 160. The application of these advanced technologies not only deepens our understanding of the immune microenvironment in NSCLC but also accelerates the development and refinement of innovative immunotherapeutic strategies. As personalized medicine continues to evolve, the design of tailored treatment regimens for specific patient subgroups is becoming increasingly feasible. By integrating comprehensive analyses of an individual's genetic background, tumor characteristics, and immune status, it is possible to more accurately predict therapeutic responses and select the most appropriate treatment options. This approach not only enhances clinical efficacy but also minimizes unnecessary side effects, offering NSCLC patients safer and more effective therapeutic choices. One of the major challenges lies in elucidating the specific roles and underlying mechanisms of TIL-Bs and autoantibodies within the immune microenvironment of NSCLC 153. Although existing studies have highlighted the critical roles of TIL-Bs and autoantibodies in tumor immunity, the inherent complexity and heterogeneity of the tumor microenvironment necessitate more granular analyses of their specific functions. Moreover, achieving precise regulation of these immune components, while minimizing the risk of autoimmune reactions or adverse side effects, remains a key challenge in translating these findings into clinically applicable therapies.

## 5. Emerging research directions on TIL-Bs and autoantibodies in NSCLC

Emerging research on TIL-Bs and autoantibodies in NSCLC heralds a deeper understanding of the TIME and the development of novel therapeutic strategies. With continued advances in immunology, molecular biology, and bioinformatics, researchers are increasingly elucidating the complex roles of TIL-Bs and autoantibodies in tumor immune evasion, anti-tumor immune responses, and immune regulation 10, 83. These advancements not only offer the potential for more personalized treatment strategies for NSCLC patients but also establish a solid foundation for optimizing immunotherapy and advancing novel drug development. Future research is expected to increasingly leverage high-throughput sequencing technologies and single-cell analysis to achieve more precise characterization of TIL-Bs and their interactions with tumor cells 161. This includes detailed investigations into TIL-B diversity, subtype classification, and functional dynamics under varying tumor microenvironmental conditions. Such studies are anticipated to uncover novel immunotherapeutic targets and signaling pathways, thereby providing critical mechanistic insights to inform the development of TIL-B-oriented therapeutic strategies 162. Research on autoantibodies is also expected to enter a new phase of development. Future work will focus on systematically identifying and functionally validating autoantibodies associated with NSCLC progression, and exploring their potential applications in tumor development, diagnosis, and therapy 163. In addition, in-depth investigation into the specific mechanisms by which autoantibodies function in tumor immune responses will support the development of autoantibody-based therapeutic approaches, such as vaccine therapy and passive immunotherapy, as well as the use of autoantibodies as biomarkers for tumor monitoring and prognostic evaluation 164. At the same time, research will further explore the interactions between TIL-Bs, autoantibodies, and other immune cells and their secreted factors to reveal their integrated effects in regulating the TIME 165. Exploration in this field is expected to promote the development of multimodal therapeutic strategies, such as combinations of ICIs, targeted therapies, and chemotherapy, to achieve more effective treatment of NSCLC. Future research directions on TIL-Bs and autoantibodies in NSCLC will focus on gaining a deeper understanding of their complex roles in tumor immunity and developing new diagnostic tools and therapeutic approaches based on these insights. As this research progresses, it is expected to offer more precise and effective treatment options for NSCLC patients, thereby improving survival outcomes and quality of life.

### 5.1 Novel immune targets and immunotherapeutic strategies

In the field of NSCLC treatment, future research roadmaps will focus on the discovery of new immune targets and the development of innovative therapeutic strategies, aiming to fully harness and regulate the patient's own immune system to combat tumors. This research direction encompasses not only in-depth investigation of the complex interactions within the TIME but also the optimization of existing immunotherapies and the development of new treatment approaches. First, future studies will intensify efforts to identify and validate novel immunotherapeutic targets in NSCLC patients. This includes detailed mechanistic investigations into the roles of TIL-Bs and autoantibodies in tumor immune evasion and immune surveillance. Through high-throughput sequencing, single-cell analysis, and functional genomics, researchers can systematically identify key molecules and signaling pathways that influence TIL-B function and autoantibody production, thereby revealing novel immunoregulatory targets 166, 167. Second, based on these newly discovered targets, research will focus on developing more specific and less toxic immunotherapeutic agents and strategies. This may involve the design of next-generation ICIs to activate or enhance the antitumor activity of TIL-Bs; the development of vaccines targeting specific autoantibodies to promote immune responses against tumor-associated antigens; and the use of gene-editing technologies to engineer Chimeric Antigen Receptor B cells (CAR-Bs). These modified B cells are designed to act as 'living factories,' continuously secreting high-affinity tumor-specific antibodies directly into the TME, representing a cutting-edge therapeutic frontier 168. In addition, the development of combination therapies will be a key focus of future research. By integrating novel immunotherapies with conventional chemotherapy, radiotherapy, or targeted therapies, a multifaceted attack on tumors can be achieved, improving treatment efficacy while minimizing damage to healthy tissues. At the same time, the design of personalized treatment strategies—based on each patient's genomic profile, TIME, and immune status—will be essential to increasing the likelihood of therapeutic success. Finally, as immunotherapy research advances and new technologies are implemented, future treatment strategies will place greater emphasis on patient quality of life and long-term survival. This will require ongoing refinement of therapeutic regimens to minimize treatment-related adverse effects, as well as the development of novel tools for treatment monitoring and response evaluation to ensure that patients receive the most effective care.

### 5.2 Technological innovations driving advances in TIL-B and autoantibody research

Technological innovations and methodological advancements have played a crucial role in driving research on TIL-Bs and autoantibodies, particularly in the field of NSCLC immunotherapy 117.

With the development and application of high-throughput sequencing technologies, single-cell analysis, immunohistochemistry, and mass spectrometry imaging, researchers can now explore the biological characteristics and functions of TIL-Bs and autoantibodies with unprecedented depth and precision, thereby accelerating our understanding of their roles in tumor immunity. High-throughput sequencing technologies, including RNA sequencing, whole-genome sequencing, and BCRs sequencing, enable comprehensive analysis of the mechanisms underlying TIL-B and autoantibody generation 152, 169. These technologies can reveal the diversity and evolution of tumor-specific BCRs and autoantibodies, laying the foundation for identifying novel immune targets and therapeutic strategies 60. Single-cell analysis technologies have further advanced this field. By analyzing gene expression and functional states at the single-cell level, researchers can uncover the heterogeneity and dynamic changes of TIL-Bs under different tumor microenvironmental conditions, as well as their interactions with tumor cells and other immune cells. Such insights are critical for understanding the specific roles of TIL-Bs in tumor immune responses and provide a scientific basis for personalized immunotherapy. The application of immunohistochemistry and mass spectrometry imaging has provided powerful tools for investigating the distribution, localization, and abundance of TIL-Bs and autoantibodies within tumor tissues. These technologies can visually demonstrate the spatial distribution and tissue architecture of TIL-Bs within the TIME, as well as the differential expression of autoantibodies in tumor versus normal tissues, offering important information for evaluating immune cell infiltration and immunotherapy response. In addition to these technological advancements, artificial intelligence (AI) and machine learning have introduced new opportunities for data analysis and interpretation in TIL-B and autoantibody research 170. By analyzing large-scale datasets, AI can help identify complex patterns and correlations, predict immunotherapy responses and patient prognosis, and ultimately guide more effective therapeutic decision-makin.

## 6. Conclusion

In the study of immune regulation in NSCLC, the investigation of TIL-Bs and autoantibodies has opened new avenues for understanding the complexity of the TIME. These immune components not only play pivotal roles in tumor recognition, cytotoxicity, and immune evasion, but also offer a solid biological foundation and translational potential for the development of novel diagnostic tools, monitoring strategies, and immunotherapeutic approaches. Recent research on TIL-Bs has highlighted their beneficial functions in NSCLC, including direct tumor cell targeting through antibody secretion, enhancement of T cell responses via antigen presentation, and modulation of the TIME through cytokine production 116, 171. These insights broaden our understanding of the multifaceted roles of B cells in tumor immunity and provide a rationale for incorporating B cell-focused strategies in the design of next-generation immunotherapies. The study of autoantibodies has further deepened our knowledge of tumor-associated immune responses, particularly regarding their utility as biomarkers and therapeutic targets. Autoantibodies not only reflect the immunological status of the tumor environment but may also signal the presence of specific tumor antigens, thereby enabling early detection and treatment monitoring. Moreover, functional analyses of autoantibodies have revealed new pathways for developing targeted and low-toxicity immunotherapies. Despite these advances, substantial challenges remain—such as how to precisely modulate TIL-B and autoantibody activity, mitigate the risks of autoimmunity, and effectively translate preclinical findings into clinical applications. Future research must delve deeper into the mechanistic underpinnings of these components and innovate new technologies to overcome current limitations, thereby unlocking their full therapeutic potential in NSCLC. In conclusion, this review proposes a conceptual shift: moving beyond viewing B cells as mere bystanders to recognizing the B-cell-autoantibody axis as a central driver of NSCLC immunity. We highlight that the controversy regarding the "dual roles" of B cells is not contradictory but contextual—dependent on TLSs maturation and cytokine milieus. Future breakthroughs will rely on integrating AI-driven profiling of autoantibody repertoires with spatial transcriptomics to stratify patients. Ultimately, translating these insights into next-generation therapies—ranging from CAR-B cells to microbiome-modulated B-cell vaccines—holds the promise of overcoming resistance to current checkpoint blockades, heralding a new era of B-cell-based precision immunotherapy.

## Figures and Tables

**Table 1:** Summary of clinical studies evaluating the prognostic value of TIL-Bs

Year	Author	Tumor Subtypes	Stage	Number of Patients	Detecting Methods	B Cell Marker	Prognostic Value	Prognostic Value
2001	Pelletier et al.^120^	NSCLC	Stage I-III	113	IHC	CD20	Positive	The presence of CD20^+^ B cells (p = 0.04) in the peritumoral region of NSCLC was a positive prognostic factor. This relation was especially strong for non-LUSC (p < 0.001).
2008	Dieu-Nosjean et al.^121^	LUAD, LUSC	Stage I-II	74	IHC	CD20	Neutral	B cells were also not associated with patient outcome (OS, DSS, or DFS).
2008	Al-Shibli et al.^122^	NSCLC	Stage I-IIIA	335	IHC (TMA)	CD20	Positive	In univariate analyses, increasing numbers of epithelial CD20^+^ (p = 0.023), stromal CD20^+^ (p < 0.001), and stromal CD4^+^ (p < 0.001) cells correlated significantly with an improved DSS.
2012	Schmidt et al.^123^	NSCLC	Stage I-IV	196	mRNA-seq	B-cell 60 genes	Positive	The B-cell metagene was significantly associated with longer survival time in the univariate (p < 0.001) and multivariate (p = 0.032) Cox regression models.
2013	Suzuki et al.^124^	LUAD	Stage IA-IB	478	IHC	CD20	Neutral	In stage I LUAD, neither tumoral (p = 0.417) nor stromal (p = 0.389) CD20^+^ B-cell infiltration demonstrated significant prognostic value for 5-year RFS.
2014	Mount et al.^125^	LUSC	Stage I-II	107	Microarray	B-cell 24 genes	Positive	B-cell-related genes were predominantly found in early-stage LUSC (OS, p < 0.001).
2015	Hern´andez-Prieto et al.^126^	NSCLC	Stage I-II	TS: 84 VS: 162	Microarrayor IHC	50-gene signature, CD20	Positive	A 50-gene signature (p < 0.0001) and CD20 (p = 0.05) were predictors for RFS in stage I/II NSCLC.
2015	Schalper et al.^127^	LUAD, LUSC	Stage I-IV	YTMA79: 202 YTMA140: 350	IHC (TMA)	CD20	Positive (YTMA79) Neutral (YTMA140)	In the YTMA79 cohort, Higher CD20^+^ B cell infiltration was significantly associated with longer OS (HR = 0.523, p = 0.004). In the YTMA140 cohort, Increased CD20^+^ B cell count showed no significant association with improved OS in NSCLC (HR = 0.887, p = 0.447).
2016	Kinoshita et al.^128^	NSCLC	Stage I-III	218	IHC	CD20	Positive (LUAD)	Multivariate analyses identified low accumulation of CD20^+^ B cells as an independent poor prognostic factor in LUAD for RFS (HR: 1.71, p = 0.004) .
2016	Kurebayashi et al.^129^	LUAD	Stage I-IIIA	111	IHC	CD20, CD138	Negative	The infiltration of B cells (interfollicular) and plasma cells (interfollicular and parafollicular) in the cancer stroma was significantly associated with poorer DFS.
2016	Iglesia et al.^130^	LUAD, LUSC	Stage I-IV	504	mRNA-seq	B-cell 60 genes	Positive (LUAD)	Expression of the 60-gene signature predicted improved OS in LUAD (HR = 0.71, p = 0.00078).
2017	Faruki et al.^131^	LUAD, LUSC	Stage I-IV	933	mRNA-seq or Microarray	B-cell specific signatures	Neutral	B cell infiltration was associated with a better prognosis in LUSC, but the association was not statistically significant (HR = 0.747, p = 0.062).
2019	Edlund et al.^132^	NSCLC	Stage I-IV	Uppsala-I: 353 Uppsala-II: 352	IHC (TMA)	CD20, CD79A, IGKC	Positive	Higher lymphocyte levels of CD20 (p = 0.042), CD79A (p = 0.004), and IGKC (p < 0.001) were significantly associated with longer OS.
2020	Lee et al.^133^	LUAD	Stage I-IV	120	IHC	CD20	Positive	In univariate analysis, intraepithelial CD20^+^ B cells showed a significant positive association with OS (HR = 0.81, p = 0.0158). The significance persisted in multivariate analysis (HR = 0.83, p = 0.048).
2020	Yang et al.^134^	LUAD	Stage I	147	IHC	CD20	Positive	In univariate analysis, CD20 (HR: 0.564, p = 0.04) was associated with RFS; multivariate analysis further revealed that CD20 (HR: 0.523, p = 0.024) was an independent prognostic factor for RFS.
2021	Amemiya et al.^135^	NSCLC	Stage I-IV	80	IHC	CD20	Positive	Patients with high CD20^+^ TILs in NSCLC had significantly improved 5-year OS (58.1% vs 34.2%, p = 0.036) and RFS (44.3% vs 17.9%, p = 0.003) compared to those with low CD20^+^ TILs.
2021	Ku et al.^136^	NSCLC	Stage IV	100	IHC	CD20	Positive	In univariate analyses, higher B-cell density (p = 0.0337) in immune cell proportions was associated with longer OS.
2021	Peng et al.^137^	NSCLC	Stage I-IIIB	681	IF	CD20	Neutral	Although intraepithelial CD20-positive B cells were associated with a tendency to prolong DFS, this association did not reach statistical significance.
2022	Song et al.^138^	LUAD	Stage I-IV	1268	scRNA-seq, bulk RNA-seq	B-cell 9 genes	Predictive value	In multivariate analysis, the signature was an independent prognostic factor (p < 0.001), with validated predictive power across six independent cohorts and clinical subgroups.
2022	Federico et al.^139^	NSCLC	Stage I-IV	ICON: 150 TCGA: 232	ICON: mIF TCGA: RPPA	CD20	Positive	In the ICON cohort, high B-cell levels correlated with longer RFS in NSCLC (HR = 0.32, p = 0.0072), while in the TCGA cohort, high CD20 expression was associated with longer PFS (HR = 0.59, p = 0.019).
2022	Li et al.^140^	NSCLC	Stage I-IV	TS: 999 VS: 570	mRNA-seq or Microarray	B-cell 28 genes	Predictive value	The B cell-related gene pairs signature serve as independent prognostic factor for NSCLC patients in both training and validation cohorts (p < 0.001, respectively).
2023	Backman et al.^141^	NSCLC	Stage I-IV	300	mIF (TMA)	CD20	Positive	B-cell density was independently associated with longer survival.
2024	Kim et al.^142^	NSCLC	Stage IV	43 (GSE126044, GSE135222)	RNA-seq	B-cell-related genes	Positive, Predictive value	Increased expression of B cell immunity-related genes is associated with better prognosis (median survival rate), and the enrichment score of all B cell subtypes in ICI responders was higher than in non-responders.

**Abbreviation:** LCLC: Large Cell Lung Carcinoma; NSCLC: non-small cell lung cancer; LUAD: lung adenocarcinoma; LUSC: Lung Squamous Cell Carcinoma; TS: Training Set; VS: Validation Set; SC: Severance cohort; TMA: tissue microarray; mIF: multiplex immunofluorescence; mRNA-seq: mRNA sequencing; scRNA-seq: single-cell RNA sequencing; RPPA: reverse-phase protein array; TCGA: The Cancer Genome Atlas; DFS: disease-free survival; RFS: recurrence-free survival; DSS: disease-specific survival; OS: overall survival; RFP: recurrence-free probability; ICI: immune checkpoint inhibitor.

**Table 2:** Comparison of phenotypes, localization, and functions between anti-tumor and pro-tumor B-cell subsets in the NSCLC microenvironment.

Feature	Anti-tumor B Cell Phenotypes (Effectors)	Pro-tumor B Cell Phenotypes (Regulators/Bregs)
Typical Markers	CD20^+^, CD19^+^, CD138^+^ (PCs), IgG^+^, CD27^+^ (Memory), CD69^+^ (Activated)	CD5^+^, CD24^hi^CD38^hi^, IgA^+^, PD-L1^+^, Tim-1^+^, IL-10^+^
Localization	Mature TLSs (GCs); Intra-tumoral regions	Immature TLSs; Dispersed in Stroma; Tumor draining lymph nodes
Key Secreted Molecules	Antibodies: IgG (High affinity, TAA-specific)	Antibodies: IgA, IgG4 (Immunosuppressive)
	Cytokines: IFN-γ, IL-12, IL-6, TNF-α	Cytokines: IL-10, IL-35, TGF-β
Effector Mechanisms	1. Efficient Antigen Presentation to T cells 2. Direct cytotoxicity via ADCC and CDC 3. Promotion of Th1 and CD8^+^ T cell responses 4. Support of TLSs structural maturation	1. Inhibition of Th1/CD8^+^ effector T cell proliferation 2. Induction of FoxP3^+^ Tregs differentiation 3. Promotion of angiogenesis and metastasis 4. Immune complex-mediated inflammation
Clinical Association	Correlated with prolonged OS and favorable response to ICI	Associated with tumor recurrence, metastasis, and resistance to Chemotherapy/Immunotherapy

**Abbreviation:** CD: cluster of differentiation; PCs: plasma cells; IgG: immunoglobulin G; TLSs: tertiary lymphoid structures; GCs: germinal centers; TAA: tumor-associated antigens; IFN-γ: interferon-γ; IL-6: interleukin-6; IL-12: interleukin-12; TNF-α: tumor necrosis factor; ADCC: antibody-dependent cell-mediated cytotoxicity; CDC: complement-dependent cytotoxicity; Th1: T helper 1 cell; OS: overall survival; ICI: immune checkpoint inhibitor; Bregs: regulatory B cells; PD-L1: programmed death ligand 1; IL-10: interleukin-10; IL-35: interleukin-35; TGF-β: transforming growth factor-β; Tregs: regulatory T cells.

## Abbreviations

NSCLC: non-small cell lung cancer
TIL-Bs: tumor-infiltrating B lymphocytes
TLSs: tertiary lymphoid structures
TIME: tumor immune microenvironment
ICI(s): immune checkpoint inhibitor(s)
Abs: antibodies
PD-1: programmed cell death protein 1
PD-L1: programmed death ligand 1
CTLA-4: cytotoxic T lymphocyte antigen-4
BCR(s): B cell receptor(s)
SHM: somatic hypermutation
CSR: class switch recombination
Tfh(s): T follicular helper cell(s)
Treg(s): regulatory T cell(s)
Breg(s): regulatory B cell(s)
NK cell(s): Natural killer cell(s)
ADCC: antibody-dependent cell-mediated cytotoxicity
ADCP: antibody-dependent cellular phagocytosis
CDC: complement-dependent cytotoxicity
IgD/M/A/IgG/IgE: immunoglobulin D/M/A/G/E
DC(s): dendritic cell(s)
FDC(s): follicular dendritic cell(s)
FRC(s): fibroblastic reticular cell(s)
HEV(s): high endothelial venule(s)
IL-2: interleukin-2
IL-6: interleukin-6
IL-10: interleukin-10
IL-15: interleukin-15
IL-21: interleukin-21
IL-35: interleukin-35
TNF: tumor necrosis factor
TGF-β: transforming growth factor-β
VEGF: vascular endothelial growth factor
IFN-γ: interferon-γ
APC: antigen-presenting cell
GzmB: granzyme B
MAC: membrane attack complex
MHC: major histocompatibility complex
OS: overall survival
DFS: disease-free survival
RFS: recurrence-free survival
DSS: disease-specific survival
PFS: progression-free survival
mRNA-seq: mRNA sequencing
scRNA-seq: single-cell RNA sequencing
RPPA: reverse-phase protein array
TMA: tissue microarray
mIF: multiplex immunofluorescence
TCGA: the cancer genome atlas
GC(s): germinal center(s)
PC(s): plasma cell(s)
SLOs: secondary lymphoid organs
CD: cluster of differentiation
CD40: cluster of differentiation 40
CD40L: cd40 ligand
MUM1: multiple myeloma oncogene 1
HSC: hematopoietic stem cell
LPC: lymphoid progenitor cell
Pro-B: progenitor B cell
Pre-B: precursor B cell
BMCA: B cell maturation antigen
MALT: mucosa-associated lymphoid tissue
TAA(s): tumor-associated antigen(s)
LUAD: lung adenocarcinoma
LCLC: Large Cell Lung Carcinoma
LUSC: Lung Squamous Cell Carcinoma
TS: Training Set
VS: Validation Set
SC: Severance cohort
RFP: recurrence-free probability
CEA: carcinoembryonic antigen
P: phosphorylation
Me: methylation
Ub: ubiquitination
Ac: acetylation
SUMO: small ubiquitin-like modifier
-S-S-: disulfide bond
BCR-seq: B cell receptor sequencing
CyTOF: cytometry by time of flight
DNA: deoxyribonucleic acid
ELISA: enzyme-linked immunosorbent assay
ELISpot: enzyme-linked immunospot
Exome-seq: exome sequencing
FACS: fluorescence-activated cell sorting
FC: flow cytometry
FISH: fluorescence in situ hybridization
HTS: high-throughput sequencing
ICC: immunocytochemistry
IHC: immunohistochemistry
IP-MS: immunoprecipitation-mass spectrometry
mIHC: multiplex immunohistochemistry
RNA-EA: RNA-based expression analysis
RNA-seq: RNA sequencing
scBCR-seq: single-cell B cell receptor sequencing
SPR: surface plasmon resonance
ST: spatial transcriptomics
V(D)J analysis: variable (diversity) joining analysis
WB: western blot
WGS: whole genome sequencing
xMAP: multi-analyte profiling
BAFF: b-cell activating factor
CXCL13: c-x-c motif chemokine ligand 13
